# Clinical exome sequencing facilitates the understanding of genetic heterogeneity in Leber congenital amaurosis patients with variable phenotype in southern India

**DOI:** 10.1186/s40662-021-00243-5

**Published:** 2021-05-06

**Authors:** Sriee Viswarubhiny, Rupa Anjanamurthy, Ayyasamy Vanniarajan, Devarajan Bharanidharan, Vijayalakshmi Perumalsamy, Periasamy Sundaresan

**Affiliations:** 1 Department of Molecular Genetics, Aravind Medical Research Foundation, Aravind Eye Hospital, Madurai, Tamil Nadu 625020 India; 2 Department of Molecular Biology, Aravind Medical Research Foundation - Affiliated to Alagappa University, Karaikudi, Tamil Nadu India; 3 Department of Paediatric and Adult strabismus, Aravind Eye Hospital, Madurai, Tamil Nadu India; 4 Department of Bioinformatics, Aravind Medical Research Foundation, Aravind Eye Hospital, Madurai, Tamil Nadu India

**Keywords:** Leber congenital amaurosis, Clinical exome sequencing, Southern India, Molecular diagnosis, Genotype-phenotype correlation

## Abstract

**Background:**

Leber congenital amaurosis (LCA), primarily characterized by retinal degeneration is the most severe form of inherited retinal dystrophy (IRD) responsible for congenital blindness. The presence of phenotypic heterogeneity makes the diagnosis of LCA challenging, especially in the absence of pronounced disease pathognomonic, yet it can be well comprehended by employing molecular diagnosis. Therefore, the present study aimed to reveal the causative mutations in ten LCA patients with variable phenotypes using clinical exome sequencing (CES).

**Methods:**

CES was performed in ten unrelated LCA patients. Ophthalmic information and family history of all patients were obtained to make a meaningful interpretation. The clinical exome data was analyzed and prioritized using a bioinformatics pipeline to identify mutations, which was further validated by Sanger sequencing. Segregation analysis was also performed on available family members.

**Results:**

CES led to the identification of causative mutations in nine LCA patients. Seven patients harbored a mutation in six LCA candidate genes, including *RPE65*, *LCA5* (*n* = 2), *CRX*, *PRPH2*, *CEP290*, and *ALMS1*, while two patients possess a mutation in *IFT80* and *RP1*, known to cause other diseases. Three novel mutations in *LCA5* (c.1823del), *CRX* (c.848del) and *CEP290* (c.2483G > T) were identified. The current study reports for the first time, a mutation in *PRPH2*, *CEP290*, and *ALMS1* from the Indian population. Additionally, we observed a novel association of LCA phenotype with *IFT80* known to cause Jeune syndrome. Based on the genetic finding, the patient AS09, who harbored a mutation in the *RP1* gene, was re-diagnosed with early-onset retinitis pigmentosa.

**Conclusion:**

In conclusion, the results underline the importance of CES in clinically diagnosed LCA patients with variable phenotypes. The correlation between mutations in candidate genes and clinical phenotypes, helps to refine the clinical diagnosis. However, molecular evaluation with a larger cohort of LCA patients is needed for better understanding of the mutational spectrum in southern India.

**Supplementary Information:**

The online version contains supplementary material available at 10.1186/s40662-021-00243-5.

## Background

Inherited retinal dystrophies (IRD) are a heterogeneous group of diseases that cause significant vision loss due to irreversible retinal degeneration. Leber congenital amaurosis (LCA) is one of the most severe and earliest forms of IRD responsible for infantile blindness with an estimated prevalence of 3 in 100,000 worldwide [1]. The incidence of LCA in the South Indian population is quite often due to consanguineous marriages and genetically isolated communities [2]. The disease begins in the first year of life with photoreceptors degeneration (rod and cone cells) and progresses through serious visual defects. The clinical hallmark of LCA includes decreased visual acuity, non-recordable electroretinogram (ERG), and sluggish pupillary response. In addition, other commonly observed clinical signs are nystagmus, Franceschetti’s oculo-digital sign, strabismus, high hyperopia, cataract, and keratoconus [3].

Although LCA is a monogenic disease, mutations in more than 29 genes have been implicated. Among these, twenty-six genes follow the autosomal recessive pattern, the classical mode of inheritance in LCA. Two genes *IMPDH1* and *OTX2,* inherit the disease in an autosomal dominant manner, while the *CRX* gene is inherited either in an autosomal dominant or recessive pattern [3, 4]. Approximately 70% of these genes contribute to non-syndromic LCA cases. Previous studies from the Indian population have observed mutations in fourteen candidate genes, including *GUCY2D*, *RPE65*, *AIPL1*, *RPGRIP1*, *LCA5*, *IQCB1*, *CRB1*, *SPATA7*, *RDH12*, *NMNAT1*, *KCNJ13*, *CRX*, *RD3*, and *TULP1* [5–7]. Few LCA-associated genes like *CEP290*, *ALMS1*, *IFT140*, and *IQCB1* also contribute to other syndromes such as Joubert syndrome, peroxisomal disease, Alstrom syndrome, Batten disease, and Senior Loken syndrome with similar ocular manifestations as observed in LCA. It mostly overlaps with a milder form of the same disease called severe early childhood-onset retinal dystrophy or early-onset retinitis pigmentosa [3, 8]. Hence, the existence of various clinical phenotypes necessitates molecular genetic testing to identify causative mutations for accurate diagnosis at the earliest instance.

Previous studies from the Indian cohort have screened very few LCA candidate genes through Sanger sequencing, homozygosity mapping, micro-array, and disease-specific targeted sequencing [5–7]. Hence, the present study employs clinical exome sequencing (CES), which targets ~ 8000 genes with known clinical implications, and thus bypass the shortcoming of other previous techniques. To our knowledge, this is the first study to use the CES approach for the molecular diagnosis of LCA patients in South India.

Therefore, the current study aimed to identify the underlying disease mutation in ten LCA patients with variable phenotypes using CES. Through the comprehensive analysis of clinical and genetic datasets, this study will contribute to the existing knowledge of genotype-phenotype associations towards LCA.

## Methods

### Ethics statement

This study was conducted in accordance with the Declaration of Helsinki and approved by the Institutional Ethics Committee of Aravind Eye Hospital, Madurai, Tamil Nadu, India (IRB2016017BAS). Written informed consent was obtained from all study patients or guardians in the case of minors or children.

### Patient recruitment

Ten unrelated individuals (AS01 – AS10) diagnosed with LCA were recruited from the Paediatric Clinic, Aravind Eye Hospital, Madurai, Tamil Nadu, India. All the patients are of South Indian origin (Tamil Nadu, Kerala, and Andhra Pradesh). Comprehensive ophthalmic examinations including visual acuity, cycloplegic refraction, color fundus photography (Topcon, Inc., Tokyo, Japan), spectral-domain optical coherence tomography (SD-OCT) and autofluorescence were performed for all study patients. ERG was recorded through the UTAS Ganzfeld-LKC technology system and Burien-Allen bipolar electrodes based on the standards of the International Society for Clinical Electrophysiology of Vision. In children below 6 years of age, ERG was performed under ketamine anesthesia.

Clinical diagnosis of LCA was based on the following criteria: i) severe visual impairment during the first year of life, especially with Franceschetti’s oculo-digital signs (eye-poking, rubbing and pressing); ii) non-recordable ERG; iii) Nystagmus or roving eye movement. A detailed pedigree was obtained as well as other particulars such as ethnic predisposition, family history and consanguinity.

### DNA isolation and CES

Genomic DNA was extracted from the peripheral blood of patients and available family members using the modified salt precipitation method [9]. CES of 10 LCA patients was performed at Medgenome, Bangalore, India. Sequencing libraries were prepared using clinical exome panel (Cev3), which covers approximately 8332 diseases causing genes, including 29 known LCA genes. Paired-end sequencing was performed to generate 2 × 150 bp reads at 100× sequencing depth using the HiSeq X Ten platform.

### CES data analysis

The pre-processing of the fastq file was performed using Cutadapt (v1.8) to exclude low-quality reads, adapters and primer sequences [10]. The pre-processed reads were aligned against the human genome reference sequence hg19 (GRCh37) using Burrows-Wheeler Aligner (BWA)-MEM (v.0.7.12) [11]. Picard tool (v.1.140; https://broadinstitute.github.io/picard/) was employed to remove PCR-duplicates. The IndelRealigner and Base Recalibrator from the Genome Analysis Toolkit (GATK, v.3.6) were used for local realignment in regions containing potential indels and recalibrating the base quality scores of all reads. Both the GATK Haplotypecaller and UnfiedGenotyper were used for variant calling [12]. Then, the variants were annotated by the Variation and Mutation Annotation Toolkit (VariMAT, v.2.4.1; https://omictools.com/varimat-tool).

### Variant prioritization

Pathogenic variants were prioritized as per the ACMG (American College of Medical Genetics and Genomics) standards and guidelines [13]. Briefly, Nonsense, frameshift, canonical ±1 or 2 splice sites, non-synonymous and in-frame variants located in the exonic region were considered for variant prioritization. The annotated variants were screened against databases such as Exome Aggregation Consortium (ExAC) and 1000 Genomes to exclude variants with allele frequency > 0.01.

The non-synonymous variants were considered as pathogenic only when at least four out of five insilico functional prediction algorithms such as SIFT (https://sift.bii.a-star.edu.sg/), PolyPhen2 (http://genetics.bwh.harvard.edu/pph2/), Mutation Taster (http://www.mutationtaster.org/), Mutation Assessor (http://mutationassessor.org/r3/) and FATHMM (http://fathmm.biocompute.org.uk/) were predicted to be deleterious. The conservation tools including GERP, SiPhy and PhastCons were used to predict the impact of non-synonymous variants. HOPE (Have (y) Our Protein Explained) was used to predict the structural impact of non-synonymous variants [14].

### Validation of variants and segregation analysis

The identified pathogenic variants were validated in probands using Sanger sequencing. Forward and reverse primers for Sanger sequencing was designed using Primer-BLAST (Supplementary Table S1). The chromatograms were visualized using chromas v.2.6.6 software (http://www.technelysium.com.au/chromas.html) and the nucleotide sequence was analyzed using BLAST. The pathogenic variants were also investigated in ethnically matched control samples. Segregation analysis of variants was also performed in available family members.

## Results

### Clinical characteristics

**Fig. 1:**
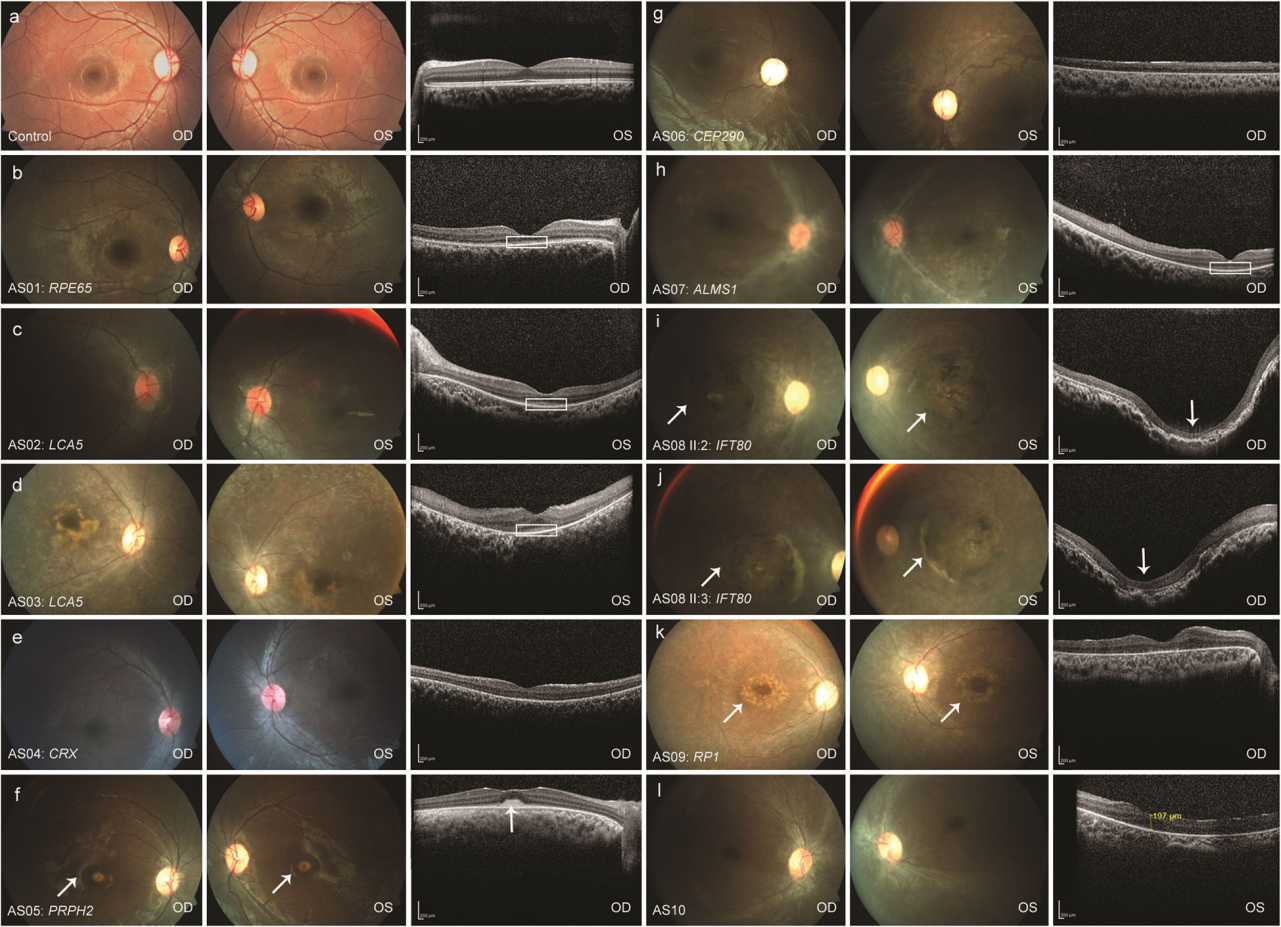
Fundus and SD-OCT of study patients. Fundus presentation ranges from greyish desaturated background to pigmentary retinopathy. **d**, **g**, **i** and **j** Patients AS03, AS06, AS08 II:2 and AS08 II:3 affected by *LCA5*, *CEP290* and *IFT80* were noted with marbled fundus. **i** and **j** AS08 II:2 and AS08 II:3 also had macular coloboma indicated by an arrow. **f** Yellow vitelliform, egg yolk like well-circumscribed lesion centered at the fovea less than 1/3 of disc with central hyperpigmented spot was observed in patient AS05 with *PRPH2* mutation indicated by an arrow. **k** white arrow indicates the Bull’s eye macula in patient AS09 carrying the *RP1* mutation. The accompanying SD-OCT revealed normal retinal architecture in patients with *RPE65*, *LCA5* (AS02) and *PRPH2*, whereas other patients had a lack of lamination or distorted retina of variable thickness resembling an immature retina. **b** – **d** and **h** Patients AS01, AS02, AS03 and AS07 affected by *RPE65*, *LCA5* and *ALMS1* mutations showed preserved outer retinal layer outlined by white lines. **f** White upper arrow specifies the vitelliform lesions at the macula in patient AS05 affected by *PRPH2* mutation. **i** and **j** AS08 II:2 and the twin AS08 II:3 presented with crater-like depression indicated by a white down arrow. Further information on these patients are described in Table 1

**Fig. 2:**
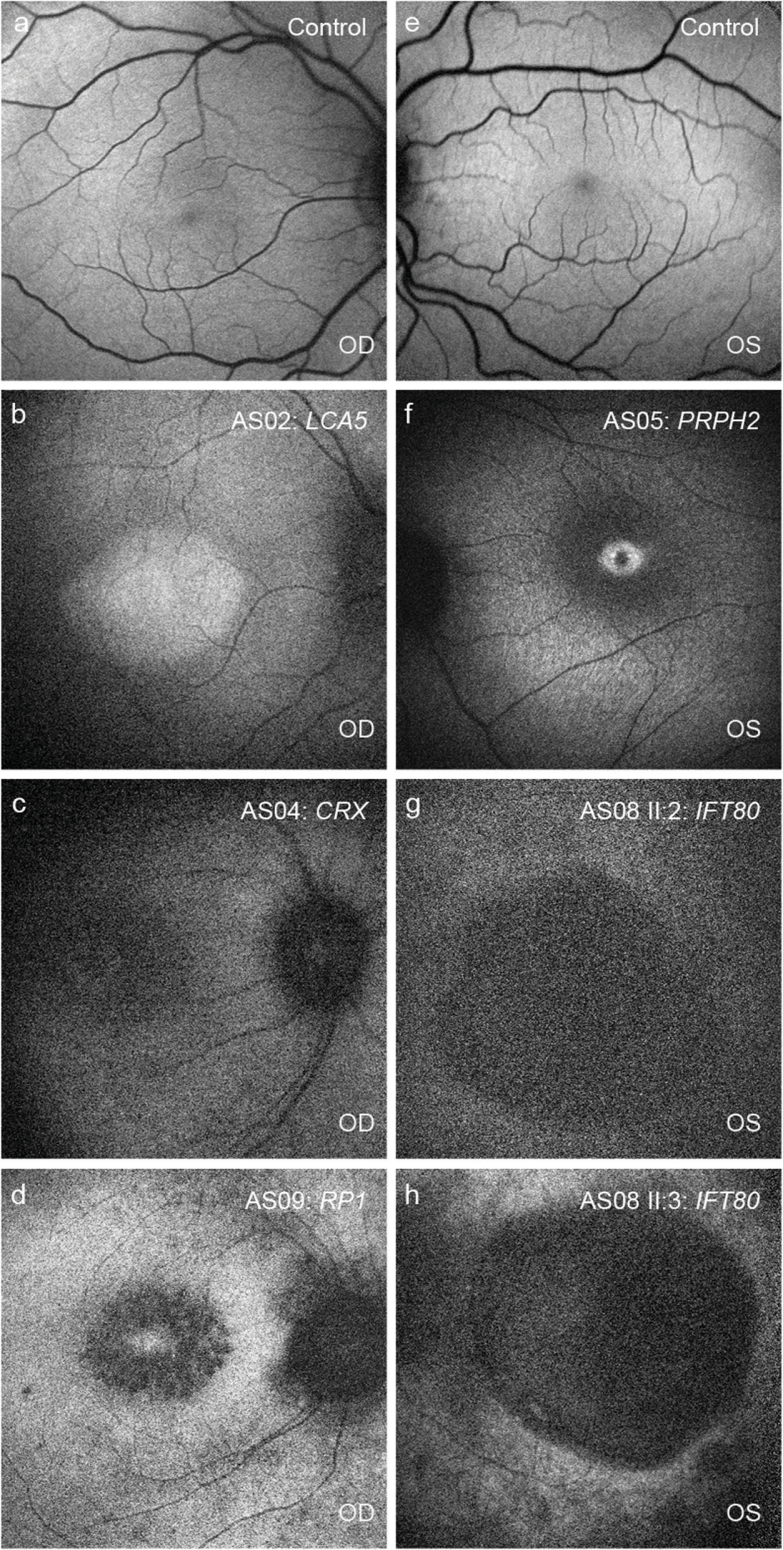
Fundus Autofluorescence photographs of some patients. Autofluorescence imaging was performed in five patients. Deviation from normal was noted in all patients

Nystagmus or roving eye movement and the oculo-digital sign was the consistent feature observed in all patients. Based on the classification of visual acuity according to the World Health Organization’s ICD-11 (International Classification of Disease 11) 2018, seven patients were legally blind while three had severe visual impairment. Scotopic and photopic responses of ERG were non-recordable in all patients. The fundus, SD-OCT, and autofluorescence of all patients are shown in Figs. 1 and 2. Two patients (AS06 and AS10) had systemic features such as head nodding, secondary behavioral changes, kidney cyst, and ichthyosis. Table 1 summarizes the clinical findings of each patient with a respective genotype.

**Table 1:** Clinical characteristics of ten South Indian patients involved in this study

Patient ID	Genetic findings	Revised Diagnosis	Age (years)			Gender	Visual acuity (Bilateral)	Refraction (Bilateral)	Clinical Presentation			ERG	Fundus	SD-OCT	Autofluorescence	Other Systemic problems
			Onset	Diagnosis	Current				Nystagmus	Oculodigital Sign	Others					
AS01	*RPE65*	LCA	1	1	8	F	6/24	LH	Horizontal jerky	Absent	Large angle exotropia, Night blindness	Non-recordable	MAA, ILM Wrinkling	NRA, Well preserved central IS/OS junction with peripheral disruption	NA	
AS02	*LCA5*	LCA	0.67	1	7	M	6/24	MH	Horizontal jerky	Present	Exotropia, Night blindness, Photophobia	Non-recordable	MAA, Pigmentary retinopathy	NRA, Well preserved central outer retinal layers, Loss of IS/OS peripheral to macula	Increased auto-fluorescence at macula	
AS03	*LCA5*	LCA	1	1	11	F	LP+	MH	Roving eye movement	Present	Photophobia, Posterior subcapsular cataract	Non-recordable	Thread like arteries, Marbled fundus, Macular atrophy	Thick distorted retina, Centrally preserved outer retinal layers	NA	
AS04	*CRX*	LCA	0.75	0.75	6	M	LP+	MH	Roving eye movement	Present	Large angle exotropia	Non-recordable	MAA, GDA	Thin distorted retina, Complete loss of outer retinal layers	Slightly reduced	
AS05	*PRPH2*	LCA	1	2	8	F	6/60	LH	Multiplanar	Present	Variable angle exotropia	Non-recordable	Yellow vitelliform lesion at fovea with central hyper pigmented spot	NRA, Hyper reflectivity of the vitelliform substance from the subretinal space	Ring of increased auto-fluorescence at macula and speck of decreased auto-fluorescence at center	
AS06	*CEP290*	LCA	0.5	0.5	7	F	LP+	HH	Roving eye movement	Present	Cortical cataract	Non-recordable	Disc pallor, Thread like arteries, Marbled fundus	Thin distorted retina	NA	Head titubation, Developmental delay, Kidney failure
AS07	*ALMS1*	LCA	1	1	6	M	LP+	HH	Horizontal pendular	Absent	Photophobia	Non-recordable	Thread like arteries, GDA, ILM wrinkling	Thick distorted retina, Central preservation of outer retinal layer, Loss of IS/OS peripheral to macula	NA	
AS08	*IFT80*	LCA	1	2	11	F	HM	MH	Multiplanar	Present	Photophobia	Non-recordable	Disc pallor, Thread like vessels, Marled fundus, Macular coloboma	Atrophied neurosensory retina, Complete loss of outer retinal layers, Crater like macular depression	Decreased auto-fluorescence at macula	
AS09	*RP1*	Early-onset retinitis pigmentosa	1.75	2	13	F	HM	LH	Multiplanar	Present	Posterior subcapsular cataract	Non-recordable	Disc pallor, MAA, Bulls eye macula, ILM wrinkling	Thick distorted retina	Decreased auto-fluorescence at macula and specks of increased auto-fluorescence at center	
AS10	No variant of interest	LCA	0.5	2	10	M	HM	M	Vertical jerky	Absent	Exotropia, Photophobia	Non-recordable	Disc pallor, Thread like arteries, GDA, Pseudohole	Thick distorted retina	NA	Dry skin, Hyperpigmented Knuckles

*M* = male; *F* = female; *LP* = light perception; *HM* = hand motion, hypermetropia classified based on refractive error; *LH* = low hypermetropia (−0.25 D to + 2.75 D); *MH* = moderate hypermetropia (+3.00 D to +5.00 D); *HH* = high hypermetropia (> + 5.00 D); *M* = myopia; *MAA* = mild arteriolar attenuation; *GDA* = greyish desaturated appearance; *ILM* = inner limiting membrane; *NRA* = normal retinal architecture; *IS/OS* = inner segment/outer segment; *NA* = not available

### Pedigree

**Fig. 3:**
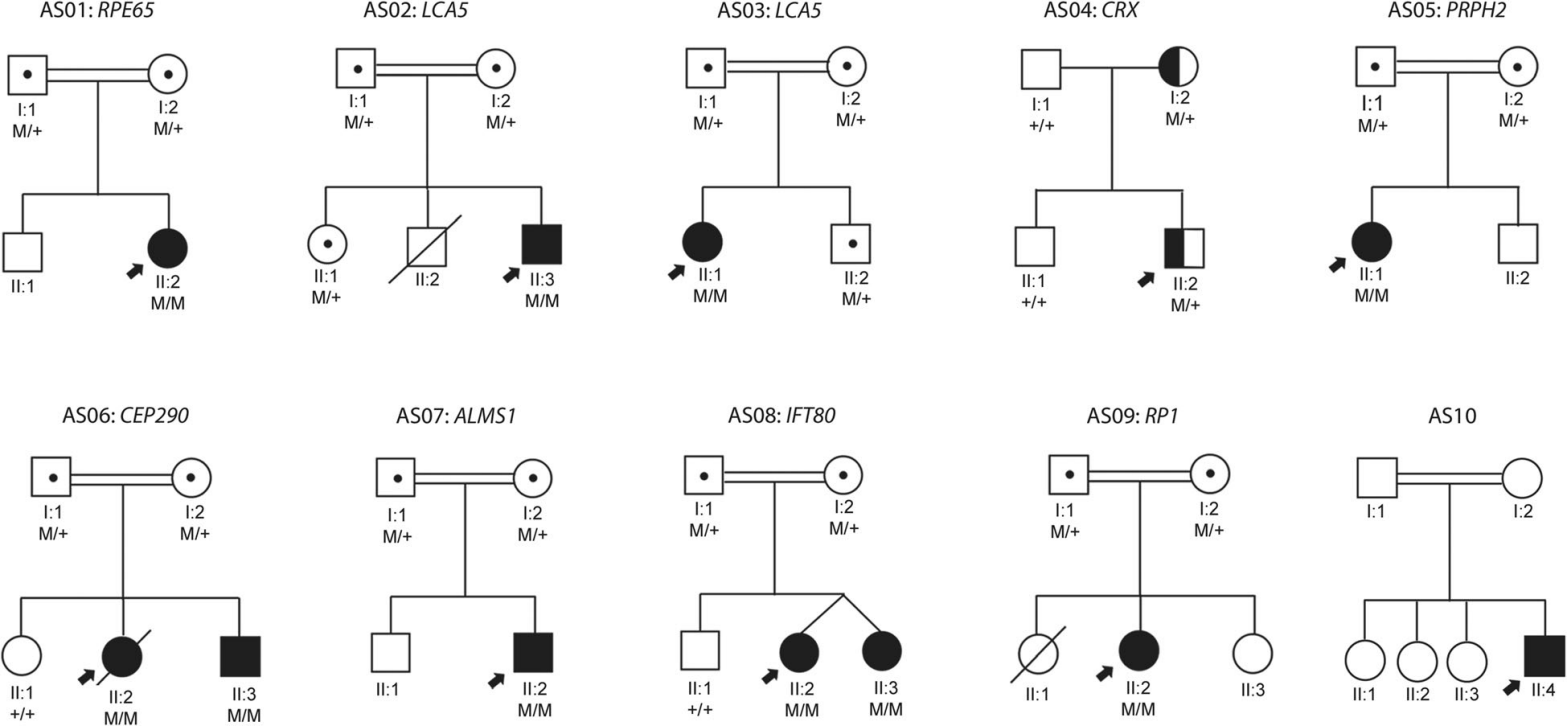
Pedigree of ten unrelated patients involved in the study. Solid symbols with an arrow – probands; Solid symbols (M/M) – homozygous affected; half-filled symbols (M/+) – heterozygous affected; dotted symbols (M/+) – carrier; unfilled symbol (+/+) – Wild type; Slash through the symbol – deceased, Consanguinity represented by the double line

Except for patient AS04, all other patients had suspected autosomal recessive pedigree due to the consanguinity and absence of consecutive generation disease. Patient AS04 may have an autosomal dominant pattern as the patient’s mother had a history of impaired vision while the father and younger sibling are normally sighted. The pedigree of all patients is shown in Fig. 3.

### Data analysis

On average, 7.5 GB of data were generated per exome, of which 95% is above Q30. Each exome contains approximately 49,000,000 reads, while an average of 47,000,000 reads remained after adapter trimming. Overall, 99.97% of reads aligned with human reference genome hg19, and 94% passed alignment. Among these mapped reads, around 86% of reads located in the target region with a sequence depth ranged from 99 to 139.09X.

Variant calling attained a total of 31,000 variants, including 30,700 substitutions and 300 indels per sample. Almost 17,000 variants were filtered by excluding variants in noncoding regions. Except for synonymous and UTR (untranslated region), nearly 6000 variants were considered for further analysis. We retained around 600 variants (100 indels and 500 substitutions) with MAF ≤ 0.01. LCA can inherit by either autosomal recessive or dominant patterns, so both homozygous and heterozygous variants were considered for analysis. Among the 500 substitutions, 50 variants were predicted to be potentially pathogenic by functional prediction and conservation tools. From the 150 variants (100 indels and 50 substitutions), putative pathogenic variants were identified. The overall summary of exome data and variant prioritization are shown in Supplementary Tables S2 and S3.

### Pathogenic mutations and segregation analysis

CES identified disease-causing mutations in nine (AS01 to AS09) out of ten patients, of which seven (AS01 to AS07) patients harbored mutations in the LCA-associated genes, including *RPE65*, *LCA5*, *CRX*, *PRPH2*, *CEP290*, and *ALMS1*. Among these, three mutations (AS03 - *LCA5*: c.1823del, p.Leu608TyrfsTer30; AS04 - *CRX*: c.848del, p.Met283ArgfsTer88; AS06 - *CEP290*: c.2483G > T, p.Ser828Ile) were novel. To our knowledge, this is the first report of a mutation of these LCA candidate genes, including *PRPH2*: c.629C > T, p.Pro210Leu; *CEP290*: c.2483G > T, p.Ser828Ile; and *ALMS1*: c.11310_11313del, p.Glu3771TrpfsTer18 from an Indian population. The remaining two patients AS08 and AS09 were identified with other retinal disease genes *IFT80* and *RP1*. The mutation (AS08 - c.1936G > T, p.Val646Phe) in the *IFT80* gene has not yet been reported to be associated with the LCA phenotype. Molecular diagnosis of AS09 with *RP1* mutation (c.3751_3752del, p.Val1251PhefsTer9) led to a revision of the clinical diagnosis as early-onset retinitis pigmentosa. The details of the mutations identified in this study have been summarized in Table 2.

**Table 2:** Putative variants of nine South Indian patients identified by clinical exome sequencing

Patient ID	Gene	Chromosomal locus	Exon	Variant Class	Variants		Zygosity	MOI	Minor allele frequency		Functional Prediction Tools				ACMG Evidence	ACMG Classification	SNP id	Reference
					cDNA_Change	Amino acid_Change			ExAC	1000G	SIFT	PP2	Mutation Taster	FATHMM				
AS01	*RPE65*	1p31.2	5	Frameshift-ins	c.361dup	p.Ser121PhefsTer10	Hom	AR	NA	NA	NA	NA	D	NA	PVS1, PM2, PP3, PP5	Pathogenic	rs121918844	13,14,15
AS02	*LCA5*	6q14.1	7	Frameshift-del	c.1062_1068del	p.Tyr354Ter	Hom	AR	NA	NA	NA	NA	D	NA	PVS1, PM2, PP3, PP5	Pathogenic	NA	17,18
AS03	*LCA5*	6q14.1	9	Frameshift-del	c.1823del^a^	p.Leu608TyrfsTer30	Hom	AR	NA	NA	NA	NA	D	NA	PVS1, PM2, PP3	Pathogenic	NA	this study
AS04	*CRX*	19q13.33	4	Frameshift-del	c.848del^a^	p.Met283ArgfsTer88	Het	AD	NA	NA	NA	NA	D	NA	PVS1, PM2, PP4	Pathogenic	NA	this study
AS05	*PRPH2*	6p21.1	2	Missense	c.629C > T	p.Pro210Leu	Hom	AR	NA	NA	D (0.03)	PoD (0.847)	D	D	PM1,PM2, PP3, PP5	Likely Pathogenic	rs61755798	24
AS06	*CEP290*	12q21.32	23	Missense	c.2483G > T^a^	p.Ser828Ile	Hom	AR	NA	NA	D (0)	PoD (0.731)	D	T	PM2, PP3	Uncertain significance	NA	this study
AS07	*ALMS1*	2p13.1	16	Frameshift-del	c.11310_11313del	p.Glu3771TrpfsTer18	Hom	AR	1.67E-05	NA	NA	NA	D	NA	PVS1, PM2, PP3, PP5	Pathogenic	rs747272625	this study
AS08	*IFT80*	3q25.33	18	Missense	c.1936G > T	p.Val646Phe	Hom	AR	5.07E-05	NA	D (0)	PrD (0.927)	D	D	PM2, PP3	Uncertain significance	rs752617502	this study
AS09	*RP1*	8q12.1	4	Frameshift-del	c.3751_3752del	p.Val1251PhefsTer9	Hom	AR	8.24E-06	NA	NA	NA	D	NA	PVS1, PM2, PP3	Pathogenic	rs745640645	this study

*Hom* = homozygous; *Het* = heterozygous; *MOI* = mode of inheritance; *AR* = autosomal recessive; *AD* = autosomal dominant. *NA* = not available, SIFT (score: 1 to 0); *D* = deleterious (≤ 0.05); *T* = tolerated (> 0.05), PP2 (score: 0 to 1); *PrD* = probably damaging (≥ 0.909); *PoD* = possibly damaging (≥ 0.447); *B* = benign (≤ 0.446), mutation taster; *D* = diseases causing, FATHMM (0 to 1); *D* = deleterious (> 0.45); *T* = tolerated (< 0.45), ^a^Novel mutations

As per the ACMG guideline, six of them (AS01 to AS04, AS07, and AS09 in Table 2) were classified to have pathogenic mutations based on the combination of the following criteria’s: PVS1: Null variant in a gene where the loss of function is a known mechanism of disease, PM2: Absent or low frequency in population databases, PP3: Several computational evidence for the mutation’s deleterious effect, PP5: Reported as pathogenic by a reputable source and PP4: Patients phenotype or family history supports variant. Patient AS05 carried a likely pathogenic mutation, which follows PM1: Variant at hotspots or functional domains, PM2, PP3, and PP5. The other two (AS06 and AS08) had variants of uncertain significance.

**Fig. 4:**
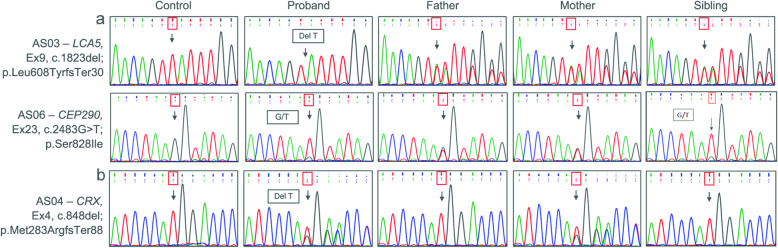
Sequence chromatogram and segregation analysis of mutations identified by this study. **a** Autosomal recessive pattern – Chromatogram shows the homozygous peak in the proband and heterozygous peak in carriers (parents and siblings). The affected sibling of AS06 (II:3) has a homozygous peak; **b** Autosomal dominant pattern of inheritance – Heterozygous peak in affected proband and mother, wild-type peak in control and other family members (father and sibling). The red box and black arrow indicate the altered nucleic acids

The identified mutations were validated in patient samples using Sanger sequencing. Chromatograms of the novel and reported mutations are shown in Fig. 4 and Supplementary Figure S1.

## Discussion

The existence of high clinical heterogeneity ensuing from intricate genetics has been demonstrated for LCA etiology. Hence, CES was performed for ten LCA patients with variable phenotypes to unravel causative mutations contributing to disease pathogenesis.

*RPE65* is one of the most common LCA candidate genes and mutation in this gene contributes to 3–16% of LCA cases worldwide [4]. Patient AS01 was identified with a pathogenic frameshift mutation in the carotenoid oxygenase domain of the *RPE65* gene, which creates a stop codon at exon 5, and thus produces a truncated protein of 129 amino acids residues. It might have partial function compared to the wild-type protein of 533 amino acids or may undergo nonsense-mediated mRNA decay. This category of gene mutations causes a deficiency of 11-cis-retinal especially in rods compared to cones, leading to nyctalopia [3, 8]. Patients with *RPE65* mutation also exhibit some extent of improvement in visual acuity over the first decade of life, but it will eventually deteriorate in the later decades [3]. Our patient also had a history of nyctalopia and showed transient improvement in visual acuity, consistent with earlier reports. The same mutation was formerly identified in South Indian LCA patients among many other mutations identified at this c.361 codon position, which makes it a hot spot for Indian LCA patients [5, 7, 15].

*LCA5* consists of 697 amino acids, which encodes highly conserved ciliary protein Lebercilin. Despite its wide expression in human tissues, *LCA5* mutations are restricted to only cause retinal dysfunction with a prevalence rate of 1–2% [4]. The majority of mutations reported with the *LCA5* phenotype are null mutations [16]. Our study identified different homozygous null mutations in two unrelated patients AS02 [17, 18] and AS03. Patient AS02 below 10 years of age showed mild improvement in vision, while patient AS03 was legally blind at 11 years of age. It was reported that patients with *LCA5* mutations showed improvement in vision and eventually decline after the first decade of life [19]. Even though Patient AS02 showed normal macula in fundoscopy and preserved central macular outer retinal layer in optic coherence tomography, increased autofluorescence at the macula was observed, suggesting increased metabolic activity of the retinal pigment epithelium. A marbled fundus was observed in patient AS03, as seen in the *CEP290*-related LCA phenotype. Patient AS03 developed a posterior subcapsular cataract, reported as a common feature of patients with *LCA5* mutations [16].

*CRX* (cone-rod homeobox) is often reported to cause LCA in an autosomal dominant pattern [20]. The present study also provides evidence by identifying a novel heterozygous frameshift mutation in patient AS04. Segregation analysis revealed the same mutation in the affected mother, while absent in normally sighted father and younger sibling, indicating that the patient inherited a disease-related mutation from his mother. *CRX* consists of 299 amino acid residues. Whereas in the proband AS04, frameshift shift mutation in exon 4 removes the native stop codon, as a result of which a larger open reading frame consisting of 369 amino acids is produced. Earlier reports on *CRX* mutations in the index cases have shown thinned, abnormal lamellar structure, and macular atrophy without noticeable signal of inner and outer segments junction in SD-OCT [21]. Similarly, patient AS04 had a lack of lamination and complete loss of the outer retinal layer.

Individuals affected by *PRPH2* mutation are known to have pattern dystrophy (butterfly-shaped pigment dystrophy and Adult-onset foveomacular vitelliform dystrophy) with a broad spectrum of clinical appearance, LCA and retinitis pigmentosa [22, 23]. Patient AS05 carried a reported missense mutation in the cytoplasmic domain of *PRPH2* [24]. HOPE predicted that the wild-type residue proline is very rigid and required special conformation of the protein backbone. Thus, the mutation at that position might affect protein function by disrupting special conformation [12]. The vitelliform lesion is the most commonly encountered clinical presentation of adult-onset foveomacular vitelliform dystrophy and LCA, due to *PRPH2*-mediated phenotypes [22, 23]. In this study, patient AS05 also demonstrated early retinal defects with vitelliform lesions, consistent with the abovementioned studies.

Interestingly, CES analysis of patient AS06 revealed a novel homozygous missense mutation in the *CEP290* gene. The HOPE tool has predicted that the mutation is present in the conserved region required for interaction with *IQCB1* (IQ Motif Containing B1). This mutation introduces more hydrophobic residues, which may affect the hydrogen-bond formation and results in loss of interactions with other molecules [12]. Mutation in *CEP290* leads to LCA, Bardet–Biedl syndrome, Senior–Loken syndrome and Joubert syndrome [25]. Unlike the Western population, where *CEP290* mutations are the most common cause for LCA with an estimated prevalence of 15–20% [4, 25], to date no *CEP290* mutations have been reported from the Indian population [13, 26]. At the age of 11 years, patient AS06 developed a cataract and presented with marbled fundus as reported in earlier studies [27, 28]. However, the presence of systemic anomalies like head nodding and secondary behavioral changes in the patient indicates an association with the abovementioned syndromes, therefore AS06 was advised to undergo complete systemic evaluation including an MRI scan and ultrasound to better understand the disease pathogenesis in this patient. However, due to kidney failure, the patient was deceased. The patient’s younger sibling (2 years) also presented with similar ocular features, head nodding and secondary behavioral changes. Since the sibling was identified with the same mutation, being strictly monitored, we hope to identify related syndromes and offer treatment early.

Mutation in *ALMS1* was reported to cause Alstrom syndrome and LCA [29, 30]. One of our patients AS07 carried a homozygous frameshift mutation in *ALMS1*. Indeed, so far *ALMS1* mutation-specific phenotypes have not been described extensively for LCA patients. However, Xu and colleagues have reported homozygous *ALMS1* null mutation in six LCA cases with early-onset retinal degeneration, visual acuity from light perception to no light, high hyperopia, roving eye movement, oculo-digital sign, undetectable ERG and tapetal fundus [30]. In agreement with this study, our patient also developed these clinical features, but the fundus revealed a pink disc, thread-like arteriolar attenuation, greyish desaturated appearance and wrinkling of the inner limiting membrane at the macula. The patient did not demonstrate any typical signs of Alstrom syndrome until now, and thus was advised to return for regular clinical assessments for any systemic abnormalities.

*IFT80* is a component of Intra Flagellar Transport complex B, which is essential for the assembly and maintenance of motile and sensory cilia [31]. *IFT80* mutants underlie Jeune syndrome, an autosomal recessive disease characterized by the constricted thoracic cage, respiratory insufficiency, cystic renal disease, polydactyl disease and retinal degeneration. Patients with Jeune syndrome are likely to develop retinal dystrophies within a few months of birth [32, 33]. In the present work, one of the twins AS08 subjected to CES was identified with a missense mutation in the *IFT80* gene. Later, the same mutation was found in another twin through segregation analysis. Both twins were presented with typical LCA clinical features as poor vision at an early stage, multiplanar nystagmus and non-recordable ERG. At 11 years, the patient observed with marbled fundus, similar to *CEP290*-associated LCA patients. The patient has not yet developed any other symptoms of Jeune syndrome except for the LCA phenotype. Many ciliopathy genes, such as *CEP290, IFT140*, and *IQCB1* initially reported to cause syndromic retinal degeneration, but were later identified as having vital roles in LCA pathogenesis as well [25, 34, 35]. Similarly, the *IFT80* gene might also be contributing to the LCA phenotype.

“The patient AS09 was initially diagnosed with LCA as she presented with poor visual acuity, multiplanar nystagmus, oculodigital sign, and non-recordable ERG at the age of 1.75 years. Interestingly, molecular diagnosis identified a homozygous frameshift mutation in exon 4 of the *RP1* gene, documented to cause retinitis pigmentosa. Previous studies have shown that the mutation in exon 4 of *RP1* causes retinitis pigmentosa in early life, as it encodes 85% of the protein [36]. Similarly, the null mutations in exon 4 of *RP1* might cause retinitis pigmentosa in the early stages of life. Thus, based on the genetic finding, the patient was re-defined with clinical diagnosis of autosomal recessive severe early-onset retinitis pigmentosa.”

The patient AS10, who was clinically diagnosed with LCA, was not identified with any underlying variant. In addition, the patient was also noted with dry, scaly skin and hyperpigmented knuckles. Whole exome or genome sequencing is required to understand the genetic cause of this disease phenotype.

## Conclusion

CES delineates the causal mutations of the LCA patients involved in this study. Our study established the involvement of new genes in LCA pathogenesis. The molecular finding also helps to assess the risk stratification due to LCA-associated syndromes. Furthermore, our results provide insight into the phenotypic features of study patients, which aid in more accurate clinical diagnosis. However, to obtain a complete picture of the disease, a large cohort of LCA patients must be evaluated and correlated with the corresponding phenotype.

## Supplementary Information

**Additional file 1: Supplementary Table S1.** Primer sequences used for the mutation validation by Sanger sequencing.

**Additional file 2: Supplementary Table S2.** Summary of clinical exome data.

**Additional file 3: Supplementary Table S3.** Overview of exclusion and prioritization of variants to obtain pathogenic variant from clinical exome data.

**Additional file 4: Supplementary Figure S1.** Sanger validation and Segregation analysis of reported mutations.

## Data Availability

All data generated during this study are included in this article and its additional files.

## Abbreviations

LCA: Leber Congenital Amaurosis
IRD: Inherited Retinal Dystrophy
CES: Clinical Exome Sequencing
ERG: Electroretinogram
SD-OCT: Spectral-domain optical coherence tomography
