# Supplementary figures and images for: Clinical exome sequencing facilitates the understanding of genetic heterogeneity in Leber congenital amaurosis patients with variable phenotype in southern India
Source: Eye Vis (Lond). 2021 May 6;8:20. doi: 10.1186/s40662-021-00243-5 (PMC8101128; doi:10.1186/s40662-021-00243-5)

**Supplementary Figure S1: Sanger validation and Segregation analysis of reported mutations**


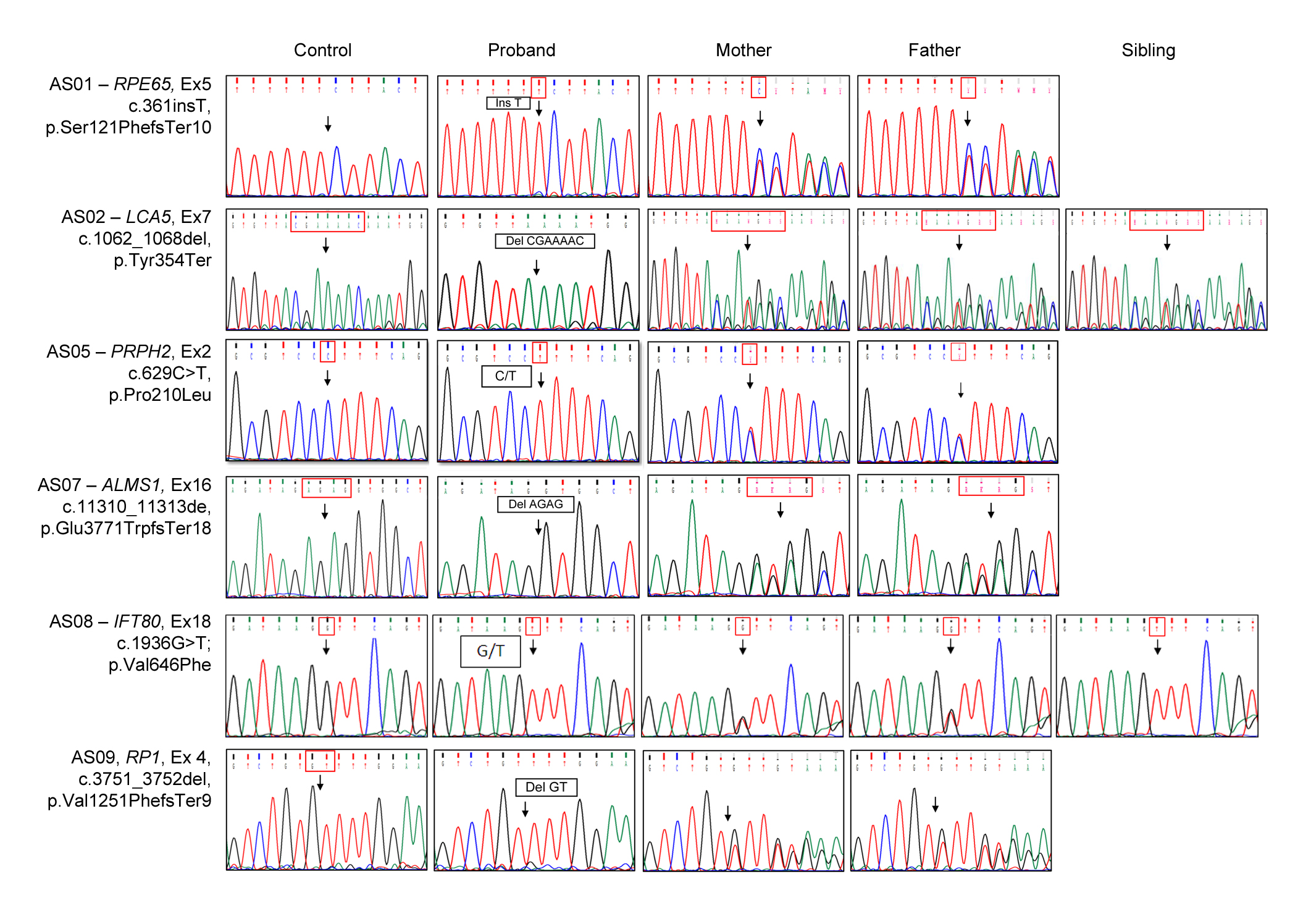

Supplement: Supplementary file 4 — Additional file 4: Supplementary Figure S1. Sanger validation and Segregation analysis of reported mutations. [file 40662_2021_243_MOESM4_ESM.docx]
